# ObStruct: A Method to Objectively Analyse Factors Driving Population Structure Using Bayesian Ancestry Profiles

**DOI:** 10.1371/journal.pone.0085196

**Published:** 2014-01-09

**Authors:** Velimir Gayevskiy, Steffen Klaere, Sarah Knight, Matthew R. Goddard

**Affiliations:** 1 School of Biological Sciences, University of Auckland, Auckland, New Zealand; 2 Department of Statistics, University of Auckland, Auckland, New Zealand; Wake Forest University Health Sciences, United States of America

## Abstract

Bayesian inference methods are extensively used to detect the presence of population structure given genetic data. The primary output of software implementing these methods are ancestry profiles of sampled individuals. While these profiles robustly partition the data into subgroups, currently there is no objective method to determine whether the fixed factor of interest (e.g. geographic origin) correlates with inferred subgroups or not, and if so, which populations are driving this correlation. We present ObStruct, a novel tool to objectively analyse the nature of structure revealed in Bayesian ancestry profiles using established statistical methods. ObStruct evaluates the extent of structural similarity between sampled and inferred populations, tests the significance of population differentiation, provides information on the contribution of sampled and inferred populations to the observed structure and crucially determines whether the predetermined factor of interest correlates with inferred population structure. Analyses of simulated and experimental data highlight ObStruct's ability to objectively assess the nature of structure in populations. We show the method is capable of capturing an increase in the level of structure with increasing time since divergence between simulated populations. Further, we applied the method to a highly structured dataset of 1,484 humans from seven continents and a less structured dataset of 179 *Saccharomyces cerevisiae* from three regions in New Zealand. Our results show that ObStruct provides an objective metric to classify the degree, drivers and significance of inferred structure, as well as providing novel insights into the relationships between sampled populations, and adds a final step to the pipeline for population structure analyses.

## Introduction

When there is a lack of free gene flow within sexual populations, neutral and selective forces will erode population homogeneity and tend to establish population structure [1], [2]. This process of subdivision will have a significant bearing on genetic diversity, local adaptation and processes such as speciation [3], [4]. When individuals sampled from discrete points (such as similar geographic locations or niches) tend to be more closely related to one another than between points, this supplies evidence of population structure. Subgroups isolated by barriers to gene flow will become increasingly differentiated by the processes of mutation, selection and drift but sufficient gene flow between subgroups will serve to homogenise groups into a single population [5]–[7]. Classic population genetics methods estimate the combined effect of these processes to infer the extent of population subdivision by analysing allele frequencies within and between sampled populations, that may or may not be differentiated [8]. Under this framework, sample locations are chosen to test factors thought to mainly define population structure, usually geographic location. The drawback of these methods is that one must *a priori* assign individuals to populations: it is conceivable that population structure exists but is missed by such *a priori* assignments because a factor other than the one considered is driving population structure. A widely used, newer and more powerful approach utilises Bayesian MCMC methods to test for population structure and dispenses with the need to assign individuals *a priori* to populations, and thus circumvents this issue [8]. These methods iteratively determine the optimal number of populations (within which there is free gene flow) given the data, and subsequently assign individuals to these inferred populations probabilistically. These methods may account for admixture, or some level of gene flow between inferred populations, in which case the proportion of each individual's ancestry in each population is estimated and thus ancestry profiles are generated for each individual [8], [9]. While these methods are powerful at determining whether structure is present, they do not allow one to determine which factors drive this structure as the current protocol relies on the subsequent subjective interpretation of plots of ancestry profiles. Simply, we propose a method to objectively analyse these inferred ancestry profiles.

structure is the most widely-used software package for Bayesian analysis and clusters individuals by attempting to create inferred populations that are in, or as close as possible to, Hardy-Weinberg equilibrium [8]. If admixture is assumed, this results in a large number of optimal configurations so structure produces ancestry profiles for each individual showing the proportion of time each individual is present in each of the inferred populations. The methods implemented in InStruct [9] also produce ancestry profiles but extend the structure algorithm and allow analysis without the assumption of Hardy-Weinberg equilibrium by calculating expected genotype frequencies based on the rates of inbreeding within each inferred population. This means InStruct is more suited to analyse populations that may be highly inbred, such as some plants and most microbes [9]. Finally, baps implements a number of novel algorithms which aim to efficiently analyze large-scale datasets to determine structure and admixture; the output of these analyses also being ancestry profiles [10], [11].

The optimal number of inferred populations may be estimated from these analyses [9], [10], [12]. The ancestry profiles for each sampled individual produced by these software packages may be examined, and a graphical overview of the patterns may be produced using distruct [13] or within baps itself. The ancestry profiles represent an objective estimation of structure and admixture within the data, and distruct visualises groupings by colouring each inferred population uniquely so that a highly structured dataset will show sampled populations mostly containing a single colour. While this approach produces a figure that is readable, it only allows a subjective visual interpretation of whether any patterns in the ancestry profiles correlate with the fixed factor of interest (e.g. geographic origin of samples). In clear-cut cases of striking population subdivision this might be sufficient, however not all datasets will show clear differentiation. Factors such as high admixture, recent divergence and inadequate sampling will create noise in the data which renders plots of ancestry profiles difficult to interpret. Presently, there is no method to objectively analyse these ancestry profiles. The method presented here addresses the interpretation of signals for population structure by analysing ancestry profiles generated by Bayesian methods, and is not concerned with the Bayesian method itself, which we consider robust.

The main aspect we consider here is how the assignment of individuals to inferred populations relates to the factor of interest. The Bayesian methods derive and assign individuals to subgroups without knowledge of the origin of individuals. Imagine one samples individuals from three geographic locations, and hence location is hypothesised to be a driver of structure. An analysis of the genotypes obtained using Bayesian methods suggest the optimal number of inferred populations is four. What does this mean? Any number of possibilities are biologically feasible: one location might harbor two or more populations, or perhaps geographic origin bears no relationship to the inferred populations, but some other factor does. The issue is that the current visualisation methods, while informative, do not allow population assignments and ancestry profiles to be objectively analysed: a subjective assessment of the plots is only possible. Our method statistically analyses these ancestry profiles and allows one to determine whether inferred population assignment and the factor of interest (e.g. origin of individuals) are significantly correlated. Having determined the extent to which a factor of interest defines observed population structure, one may then conduct finer scale analyses to ascertain the relative contribution of each sampled and inferred population to overall population structure. Which sampled and inferred populations are most differentiated or contribute the most to overall structure? We set about applying a statistical procedure which can objectively quantify the level of structure in these ancestry profiles, test the sources of structure, and determine statistical significance using a permutation approach. Our method complements visualization with distruct, adds a final step to the pipeline for population structure analyses, and allows one to analyse factors driving population structure within ancestry profiles and the extent to which these factors are explaining the variability seen within ancestry profiles as a whole.

## Methods

### Data

ObStruct directly takes structure [8], InStruct [9] and baps [10], [11] outputs from analyses that include admixture. For each individual sampled the outputs contain the proportion of ancestry in each inferred population, summing to one. A specific range of inferred populations (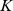) is typically run to determine the optimal value of 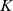 that gives the highest resolution of individuals to inferred populations. ObStruct can either use the optimal value of 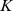 determined by InStruct using Deviance Information Criterion (DIC) or a value specified by the user. baps can estimate 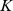 using its 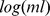 algorithm or use a value specified by the user. structure does not estimate an optimal 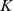 and needs a secondary method to determine the optimal 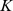 (e.g., [12]).

ObStruct tests whether the population structure represented by the ancestral profiles is correlated to the structure given by the predefined populations (sampled populations). Predefined populations are discrete sampling units within the data based on the factor of choice. For example, predefined populations in a geographic study would be different sampled regions. Predefined populations can be specified at different categorical scales to explore a single dataset in multiple ways, e.g. by language, continent, altitude, salinity, region, pH, etc. If inferred structure correlates with predefined populations, individuals within each of the predefined populations will tend to have high values of ancestry in a small number of unique inferred populations.

### The 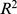 Statistic

Our aim is to determine the extent to which the factor of interest (encoded as the predefined populations) is reflected in the ancestry profiles. We use the 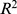 statistic to quantify this extent. Let 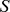 denote the number of predefined populations, and 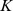 the number of inferred populations. Let 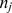 denote the number of individuals in population 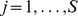, and let 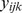 denote the ancestry of individual 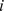 from predefined population 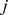 in inferred population 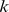. Hence, we have 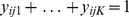. Further let 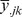 denote the proportion with which the average individual in predefined population 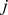 is in inferred population 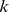 (also known as the group mean), and let 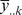 denote the proportion with which the average individual is in inferred population 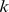 (also known as the overall mean).

Our null hypothesis states that the inferred ancestries do not reflect our predefined populations, i.e. individuals inferred to share a high proportion of ancestry (forming a population within the data) appear randomly scattered among the predefined populations or, alternatively, all individuals have equal ancestries to all inferred populations. In short, this indicates the factor of interest does not account for or drive inferred population structure. An established way of assessing how well the predefined populations are represented by the inferred populations is by evaluating the variation within and across predefined populations (e.g., [14], [15]). The sum of squares across populations (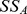) is given as follows:
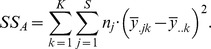



The 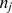 in the summation accounts for heterogeneous population sizes. The sum of squares within populations (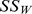) is given by:
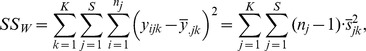



where 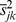 is the empirical variance of population 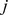 within group 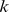. To see how much of the variability in the ancestry profiles is explained by the predefined populations, we simply compute the multiple 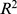 statistic:
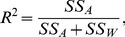
(1)


i.e., we assess how much of the total variability in the data is accounted for by grouping the data points according to the predefined populations. The 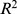 statistic is well-known and easily interpretable. A low 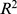 means that either the predefined populations have diverged quite recently or that there is a lot of migration and admixture between the populations. A high 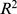 indicates strong diversification and/or population structure.

The usual way of assessing the amount of evidence against the null hypothesis is by computing a 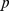-value. In classic ANOVA, one uses 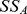 and 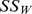 to build the 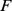 statistic which is 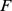-distributed with degrees of freedom corresponding to 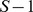 and 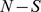, where 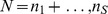. However, since we have 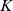 response variables this approach is no longer valid [15]. Instead, we generate a distribution for the 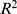 statistic under the null hypothesis using permutations of individuals (e.g., [14], [15]). If our null hypothesis is true, then the perceived similarity within predefined populations is arbitrary and permuting the ancestry profiles of the individuals should not significantly change the 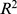 statistic. For our approach we considered 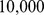 permuted datasets appropriate. The 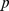-value is then the proportion of permuted datasets for which the 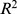 statistic exceeds the value for the initial dataset.

Since we assess the null hypothesis using the variability in the data, and the data are proportions, we have to account for heterogeneity in variance. Proportions close to 0 or close 1 tend to have a smaller variance than proportions close to 0.5. Not accounting for this can lead to observing effects that are due to the heterogeneity in the variance rather than similarities in the population structure. To address this problem we follow the common approach and use the logit transform on the proportions, i.e. we replace 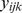 with:
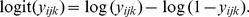



### Additional Analyses of Population Structure

#### Relative Contributions of Populations

Having established the overall level of structure within the dataset, we can apply the statistic to examine the relative contributions of each of the predefined and inferred populations to this structure. To assess the contribution we remove one predefined or one inferred population at a time and recalculate 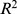 for the reduced dataset. An increase in 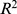 means that the removed population was more homogenised than average and thus contributed less than average to the structure in the data. A decrease in 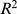, on the other hand, indicates that the removed population contributed to an increase in structure by discriminating against the other populations or showing discriminating structure itself. Such an analysis has biological relevance in determining the main drivers of population structure, e.g. whether just a few populations are giving rise to the total structure seen in the data.

#### Pairwise Comparisons of Predefined Populations

The primary units of interest are populations deriving from the predefined factor level (e.g. geographic location) so we next focus on further analysing the patterns of structure between them. This allows us to identify similarities and differences between predefined populations and test the significance of these relationships. To do this we apply the 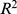 statistic calculation for all pairwise combinations of predefined populations to produce a pairwise matrix of 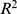 values. To access the significance of the similarities or differences between predefined populations we again carry out by permutation of ancestry profiles within each pairwise combination and correct for multiple re-sampling with Bonferroni correction.

#### Visualisation of Structure

An integral part of every statistical analysis is plotting the data to visualise the outcome of the analysis. To visualise the structure derived from the inferred populations and their relation to the predefined populations we use canonical discriminant analysis (CDA, see e.g., [14]). CDA is a method to assess and visualise the correlation between a set of response variables and a set of dummy variables coded from the factor variable. Here, each inferred population is treated as a response variable and the predefined populations compose our factor variable. The data on which the CDA is executed are the logit-transformed ancestral profiles. A CDA starts by fitting a linear model between each inferred population and the predefined population, and then combines these 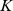 models into a single model which assesses the correlation of the inferred populations. It then suggests a set of transformed, orthogonal variables which help visualising the observed divergences in the data. The difference of a CDA to the more popular principal component analysis is the explicit inclusion of the explanatory variable in the calculations.

The output of ObStruct contains a script executable in the statistical software **R** (http://www.r-project.org), using the **R**-specific packages candisc and heplots [16]. Upon execution it will create three figures.

The first figure visualises all individuals coloured according to the predefined population they belong to. The two axes are labelled with the two variables explaining the highest proportion of variability in the data. This proportion is part of the axis label, thus providing the user with information about the amount of variability visualised in the 2D-plot. Further, the plot shows two ellipsoids centred at the hypothetical average over all data points. The inner ellipse contains approximately 50% of the individuals while the outer ellipse contains about 95% of the individuals.

The second figure summarises the information of the first by drawing 66% ellipsoids for every predefined population centred at the respective population mean. This type of plot indicates the position of the predefined populations relative to each other when given the transformed variables.

The final figure is called an HE plot. Here, the H stands for hypothesis, and E stands for error. The plot will post the same axis labels as the previous plots. However, the predefined populations are reduced to simply show their centres. In addition, inferred populations are visualised by arrows indicating their relation to the transformed variables. Possibly the most important feature of the plot are the two ellipsoids. The one labeled group indicates the range of individuals, while the red ellipsoid labeled error indicates the range of variation between the group means if predefined and inferred populations do not resemble each other. If there is no resemblance in structure the red ellipsoid will be large and potentially exceed the group ellipsoid, while a strong resemblance will lead to a small error ellipsoid within the group ellipsoid.

### Trial Datasets

To test the effectiveness of our method, we applied it to simulated and experimental data. Simulations using coalescent theory allow the demographic modeling of population structure backwards in time. This means we can directly compare the divergence of populations with known parameters with the performance of our method to describe population division processes throughout the simulation. The application of our method to experimental data then serves to illustrate the practical benefits of the method and provides further useful information on the data.

#### Simulations

The fastsimcoal software [17] was used to simulate three populations of 25,000 diploid individuals each diverging into 5 populations of 5,000 diploid individuals and then continuing to evolve without gene flow for 1,000 generations. Samples of 100 individuals from each population were taken every 50 generations starting from the initial divergence up to 1,000 generations, resulting in 21 sampling points per population. The simulation outputs were specified as 10 unlinked microsatellite loci with the geometric parameter for a Generalised Stepwise Mutation model of 0.05. Ten markers were used as a conservative measure of variation, if structure can be found in ten markers then more will only add to the power of the method. The microsatellite profiles were analysed for population structure using InStruct and structure (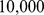 iterations of burn-in, 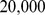 iterations of sampling, 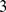 chains) with 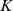 set to 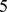 since this is the true number of populations within this dataset. The resulting ancestry profiles were used to calculate 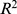 to determine whether the increasing population structure generated in the simulations was recovered with this new method of analysis.

#### Experimental Data

We chose to analyse two published datasets showing high and low levels of population structure in order to evaluate the effectiveness of our method across a range of conditions encountered in nature. The first of these datasets comprises 1,484 humans genotyped at 678 microsatellite loci in 78 worldwide populations from 7 distinct geographic continents [18]. 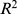 was calculated with predefined populations specified at both the continental and regional scales to determine whether greater population structure was observed at finer-scale sampling, as might be expected. The second dataset comprises *Saccharomyces cerevisiae* (a microbial sexual diploid eukaryote) isolates sampled from three regions of the North Island of New Zealand and genotyped at 9 microsatellite loci [19]. A conserved microsatellite binning procedure was applied to the data leaving a total of 179 isolates from the three sampled regions. Microbes are not constrained as heavily by body size, population density or range size as are larger organisms, leading some to hypothesise an increase in passive dispersal ability [20]. *S. cerevisiae* also has an extensive history of human association [21] which means this dataset might contain less population structure due to increased gene flow between regions due to both passive and human-mediated movement.

The human data were analysed with structure as per [18] for 10,000 iterations of burn-in and 20,000 iterations of sampling using the admixture model. 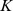 was set to 6 as this was the maximal value that was found to significantly increase the resolution of the resulting ancestry profiles in the original study. Three independent chains were run to check for convergence. Due to the highly inbred nature of *S. cerevisiae*, InStruct was run on this dataset for 100,000 iterations of burn-in and a further 100,000 iterations of sampling using the admixture model from 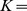 1 to 30 and 3 chains per 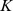. Both programs output ancestry profiles in the distruct format which ObStruct parses. To provide a visual comparison with our method, ancestry profiles were plotted using distruct [13].

### Implementation

The ObStruct method is implemented in a Perl script called ObStruct.pl which, along with documentation, is available from http://goddardlab.auckland.ac.nz/ObStruct. The script takes outputs from structure, InStruct and baps directly to calculate 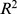 values and generates two output files: a comma-separated text file summarising the results of the 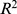 analyses, and an R-script providing the commands to visualise the relationship of predefined and inferred population structure in the data.

## Results

### Simulated Data

**Figure 1:**
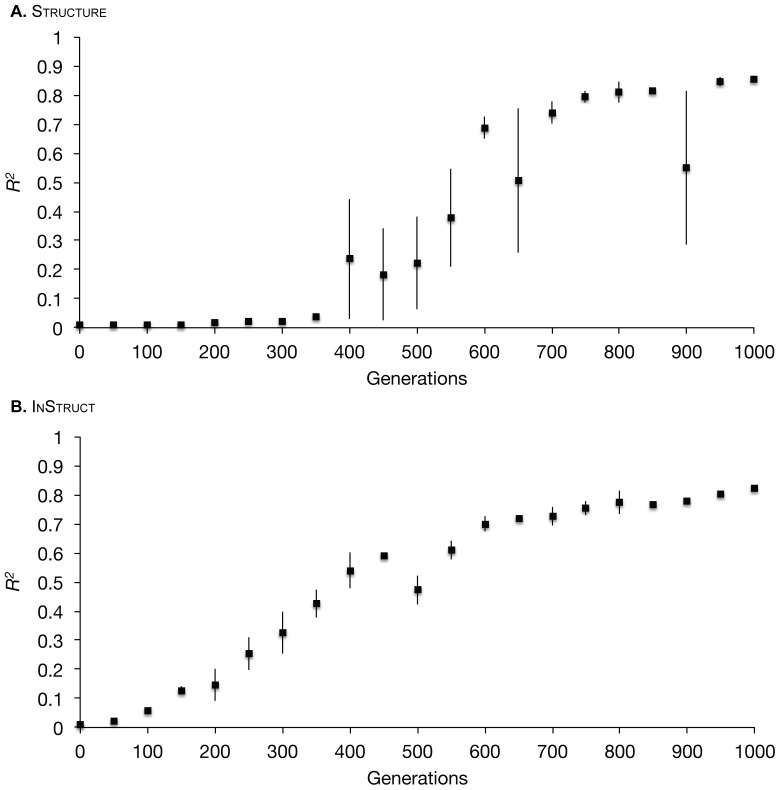
Change in population structure over 1,000 generations of simulated population divergence as inferred by (a) Structure and (b) InStruct. Error bars denote the standard error of three separate simulations for each sampling time point.

The 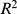 proportions generated from the simulated datasets are plotted in Figure 1 for structure and InStruct. As expected, both figures show an increase in the level of inferred population structure through time after the initial divergence event. Since no gene flow occurred between populations and the rate of mutation is the same for all populations, the only variability in the simulations should be that of sampling. 5,000 individuals are present in each population but just 100 are sampled at each time point. This variability gives rise to stochasticity in the levels of population structure which is further operated on by their analyses leading to variation in the 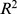 proportions, shown by the error bars.

Our analysis shows InStruct gave rise to a consistent relationship between time and structure with little variability seen within each sample for the three replicate simulations (Figure 1b). structure displayed a similar relationship but with significantly increased error around 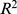 proportions (Figure 1a). The detection of significant population structure, as determined using permutation significance testing, also showed a similar pattern where significance (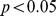) was observed for all simulations after 50 generations in the InStruct analyses while the last non-significant replicate was observed at generation 450 for the structure analyses. The gradual increase in structure reported by InStruct more accurately reflects the expectations given the parameters of the simulations, given the fact that populations are necessarily inbred due to the absence of gene flow between populations.

structure aims to maximise Hardy-Weinberg equilibrium within inferred populations whereas InStruct instead focusses on using inbreeding rates to calculate expected genotype frequencies within inferred population. The choice of software should be made based on biological information instead of statistical considerations. Nevertheless, these results clearly show that ObStruct is capable of identifying the predefined population structure in the ancestral profiles produced using both methods, if it exists.

### Results for Experimental Data

Applying the ObStruct method to experimental data allows an additional empirical analysis of population structure over the description of graphical outputs of ancestry profiles using district plots. Graphical outputs are open to interpretation whereas ObStruct provides objective insights into the resemblance between predefined and inferred population structure. We will not only mirror the conclusion of the studies from which the data are taken, but also expand upon these results and provide new insights.

#### Geographically-diverse Human Microsatellite Profiles

We recapitulated the analyses of data from [18] using structure with 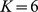. 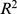 was calculated when the data were partitioned by continent (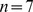) and region (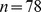) to enable analysis of structure at disparate scales. The resulting 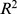 values are shown in Table 1. The structural resemblance observed is much higher when partitioned by region (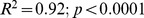) compared with partitioning by continents (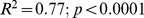). This is not surprising since we would expect individuals sampled at finer scales to be more closely related. The variability observed between the three chains run for each dataset is minimal, indicating adequate convergence of chains.

**Table 1:** 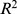
 values calculated for two experimental datasets of human and *S. cerevisiae* microsatellite profiles.

Dataset	Scale	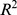
Human	Continental	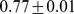 ***
	Regional	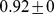 ***
*S. cerevisiae*	Regional	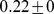 ***

The error reported is the standard error of calculating 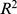 for three separate chains of each dataset.

Denotes (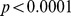).

**Figure 2.World:**
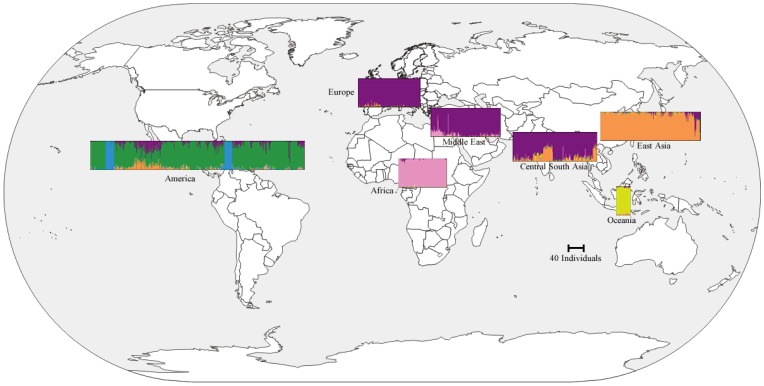
map showing distruct plots of ancestry profiles for 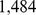 humans sampled from seven continents from [18]. Ancestry profiles were generated using structure with 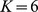.

Table 2 shows the changes to the 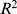 values when each predefined continental population is removed. Removing Africa reduced 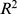 the most, meaning that this continent has the highest proportion of individuals with high ancestries to a single inferred population, and thus contributes the most to the signal for population structure. The removal of East Asia and Europe reduced the 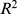 value below the overall value by a smaller margin indicating higher than average levels of structure within these continents. The Middle East and Oceania left the value of 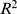 unchanged. Continents that caused 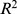 to increase above the overall value were Central South Asia and America. Removing America in particular causes a large increase in 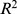 from the observed value, indicating that it is the most heterogenous continent harboring individuals with mixed ancestries. These analyses complement patterns seen in the distruct plot of this data shown in Figure 2. However, Oceania appears to be highly structured into a single unique inferred population in the plots but its removal does not alter the 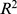 value in our analyses. The reason for this lies in this continent's small population size of 36 individuals. The calculation of 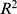 takes into account sample size and will adjust for populations with small sizes since their high structure might be due to chance. Our choice to include the Oceania data stems from the desire to use the full dataset from the original publication and show how our method deals with small population sizes.

**Table 2:** Changes from the observed 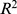 value when each continent and inferred population is removed in turn for the human dataset.

Predefined Population	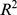	Inferred Population	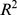
Africa	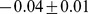	6 (Purple)	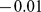
East Asia	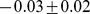	5 (Green)	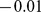
Europe	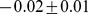	4 (Pink)	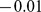
Middle East	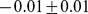	1 (Orange)	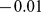
Oceania	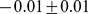	3 (Yellow)	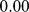
Central South Asia	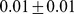	2 (Blue)	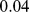
America	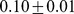		

The colours for inferred populations correspond to those seen in Figure 2. The error reported for the continents is the standard error of 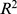 calculated for three separate chains of each dataset. No such error is reported for inferred populations because the designations for inferred populations differ between chains.

Table 2 also shows the changes in 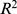 values when each of the six inferred populations is removed in turn for the continental scale. All inferred populations apart from two decrease 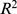 from the overall value, which indicates these inferred populations are contributing to structure within the data. Inferred population three doesn't change the 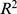 value and inferred population two increases it (shown in blue in Figure 2). Individuals with high ancestry to this inferred population come from two regional populations within the American continent. Since this inferred population only occurs in America as a subset of the overall diversity in that continent, removing it serves to increase the signal of structure from the entire American continent. This shows the diagnostic value of this technique for identifying potential sub-structure within predefined populations.

Table S1 shows 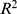 values for each pairwise combination of sampled continents. When visually compared with the distruct plot of the data (Figure 2), a number of interesting patterns emerge. First, among these are the significantly reduced 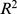 values for all pairwise combinations involving America, resulting from the high heterogeneity of ancestries within the American continent. Comparisons of the three continents with high levels of ancestry in the purple inferred population have 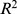 values at or below 0.3, indicating relatively little differentiation. The largest increases to 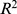 are observed in pairwise comparisons involving Africa, showing that this continent is the most distinct. East Asia shows a similar pattern to Africa but not to the same extent due to the admixture seen within it (purple bars amongst the orange). Oceania is a unique case since in the distruct plot it appears to be highly structured, but the pairwise 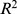 values are lower than for the similarly highly-structured Africa. This is again explained by the small sample size from the Oceania continent which means this sample is biased within any pairwise combinations by contributing less to the sum of squares across (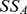). While many of the patterns observed in our analysis can be seen within the distruct plots, it is important to stress the objective nature of our analyses supporting the subjective interpretation of plotted ancestry profiles. Further, not all datasets are as clear-cut and easy to interpret as this highly-structured dataset, the next experimental dataset on *S. cerevisiae* strains illustrates this.

#### *Saccharomyces cerevisiae* Microsatellite Profiles

**Figure 3.Map:**
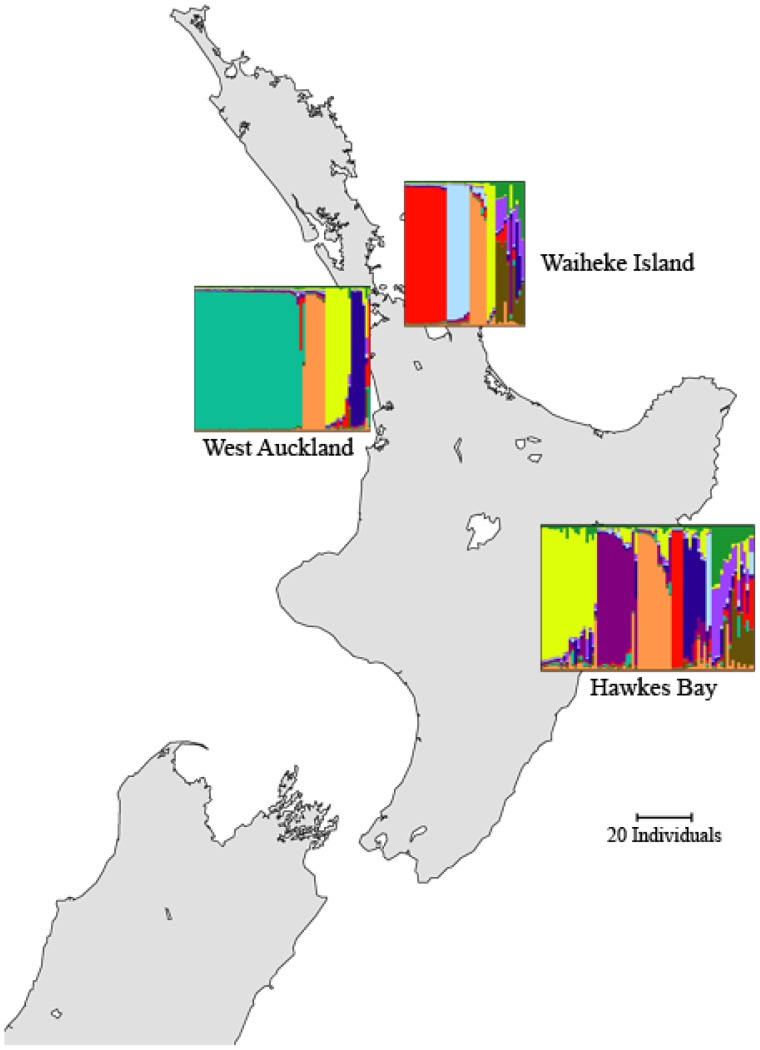
of New Zealand showing distruct plots of ancestry profiles for 179 *S. cerevisiae* sampled from three regions from [19]. Ancestry profiles were generated using InStruct with 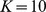.

Table 1 shows that the observed 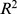 value for the *S. cerevisiae* dataset is 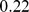 (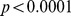). Figure 3 matches the ancestry profiles for this dataset, as generated with distruct, to the location of the sampled regions in New Zealand.

The relatively low 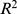 value compared to the human dataset is due to two reasons, (1) only nine loci are used in the yeast data while there were 678 loci encoding divergence in the human data, and (2) these yeast populations appear to experience more admixture than human populations. Despite this, we observe structure within inferred populations that is explained by corresponding sampled regions, i.e., unique genetic diversity is found within at least one of the sampled regions. Our in-depth analysis (Table S2) shows that removing West Auckland from the data leads to a reduction in 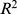 by 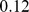, meaning that this region contains the highest proportion of individuals with high ancestries to inferred populations not seen elsewhere; the turquoise inferred population in Figure 3. Waiheke Island and Hawke's Bay contain some unique population structure as evidenced by a significant 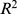 value when West Auckland is removed from the data. Unique structure is seen in the Waiheke Island and Hawke's Bay regions in the light blue and purple inferred populations within each region (Figure 3), and this structure is enough to reduce the 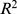 value slightly by 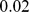 for Waiheke Island and 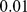 for Hawke's Bay when each region is removed from the data.

Finally, the changes to 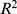 when each inferred population is removed in turn (data not shown) indicate that a single inferred population, when removed, reduces the 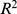 by 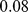 while the rest increase it by 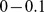. This indicates that one of the inferred populations is driving structure within the dataset, i.e. comprises a large number of individuals from a single region. This pattern is identical for all three chains run for the data. This population is in fact the turquoise inferred population seen extensively in West Auckland, shown in Figure 3.

**Figure 4.**
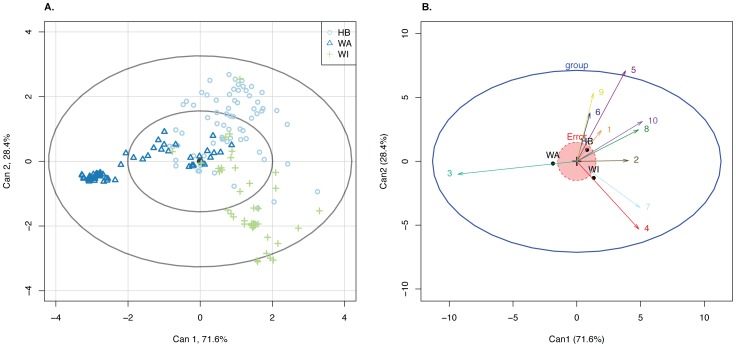
Canonical discriminant analysis on the *Saccharomyces cerevisiae* dataset. (a) Mapping the individual data according to the CDA variables. The inner ellipsoid contains 50% of all individuals, the outer ellipsoid contains 95% of all individuals. Individuals are colour- and shape-coded according to their respective sampled region. (b) The HE plot shows the relation of variation in the group means on two variables relative to the error variance. The coloured arrows indicate the position of the inferred populations relative to the axes obtained by the canonical discriminant analysis. The black points indicate predefined populations (WA  =  West Auckland; WI  =  Waiheke Island; HB  =  Hawke's Bay) while numbers at the arrows indicate inferred populations.

Figure 4 shows two of the plots ObStruct creates when applied to this dataset. We see that two transformed variables are sufficient to visualise the variation for all individuals, with the first variable covering 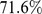 and the second the remaining 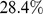. Figure 4a visualises the position of individuals to each other, coloured by their membership to a predefined population. We see that all three populations are relatively separate with West Auckland showing a large cluster of individuals separate from the rest. Figure 4b reduces the sampling populations to their center and visualises the influence of the inferred populations with arrows (inferred populations are coloured the same as in Figure 3). We see that the turquoise arrow representing inferred population 3 strongly points toward West Auckland encompassing the cluster of individuals there. The unique structure within Waiheke Island is encompassed by inferred populations 4 and 7, with the rest of the inferred populations covering the direction of Hawkes Bay. While this can also be seen in the distruct plot, the HE plot adds an extra layer by visualising the strength of resemblance through the red error circle which indicates that Hawke's Bay and Waiheke Island could be considered more similar to each other than to West Auckland, an observation that is not as obvious when looking at distruct plots.

Note that not every dataset will show enough discrimination with just two CDA variables. The variation indicated at the axis is a good indicator of how much variation has been covered by the CDA variables. If a third variable is useful, use the **R**-function heplot3d for an interactive, 3-dimensional form of the HE-plot.

## Discussion

We have presented a novel application of a classic statistical tool to analyse ancestry profiles produced from the Bayesian methods implemented in structure and InStruct. Our method analyses the signals for population structure present in these ancestry profiles and determines the extent to which inferred structure correlates with the predefined factor of interest. We believe this method is a valuable addition to the existing pipeline for analysing population structure and extends the subjective interpretation of plots of ancestry profiles. To support our position we have applied the method to three distinct datasets: analyses of simulations show the ability of ObStruct to capture information on overall structure; analyses of human data show how the method performs on a highly structured dataset; and, analyses of *S. cerevisiae* data show how the method performs on a highly admixed dataset.

The steady increase in structure through time since divergence seen in the simulation data would not be easy to determine using present methods in population genetics. Our method quickly and easily captures this information and tests significance using a permutation approach. We found that within our simulations significant population structure could be detected in all three replicates after 50 generations by InStruct and after 450 generations by structure. After 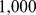 generations of divergence, analyses of outputs from structure and InStruct converged on 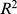 values around 0.85, although structure produced inconsistent 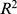 values, possibly due to the way the method attempts to cluster inferred populations based on assumptions of Hardy-Weinberg equilibrium. Testing for differences in the effectiveness of these two methods is outside the scope of this study, but these results indicate the 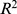 is suitable for such tests and clearly indicates the levels of population structure in datasets.

The application of our method to experimental data showed the comparability of the overall 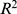 value between datasets. The highly admixed *S. cerevisiae* had a much lower 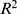 value of 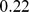 compared with 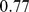 for the human data partitioned by continent, or 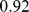 by region. Our new method allows one to test if the factor of interest correlates with inferred structure. Further, we were able to objectively determine the sources of structure within these datasets. Understanding the drivers of structure allows us to draw biologically relevant conclusions and understand the relative relatedness of sampled populations.

The absolute 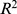 value is useful for comparing datasets but it is difficult to generalise specific 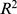 values to categorical levels of structure. Rather, we recommend in-depth exploration of the dataset by using the pairwise predefined population matrix and lists of 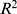 values when each predefined and inferred population is removed. It is this exploration that uncovers sources of structure within the data between specific populations, for example, it may be possible that one predefined population is very distinct from the rest of the data which itself shows high admixture. This means that the overall 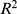 value is primarily itself only a benchmark against which to compare the changes to it from deeper tests performed by our method. Manual manipulations of ancestry profiles show that the 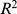 statistic is able to differentiate differences in ancestry proportions as low as 1% which makes it a sensitive measure of structure.

The method described in this work has wide-ranging applications to any field employing population genetic techniques, and we feel that this is a valuable addition to a pipeline for the analyses of population structure. An objective quantification of population structure in datasets means that disparate datasets may now be compared. This opens up the ability to conduct theoretical and practical tests on the nature of population structure and the factors that influence its inception and perpetuation. The ability to look within a dataset at the causes of structure help to determine the relative difference of populations and allows further interpretation of the data. We believe that objectively quantifying the levels of structure in data and taking into account important characteristics such as population size, number of predefined populations and statistical significance is a significant addition to the currently available analyses.

## Supporting Information

Table S1
**Pairwise matrix of** 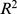 **values between continents for the human dataset.**
(PDF)

Table S2
**Pairwise matrix of** 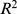 **values between regions for the** ***Saccharomyces cerevisiae*** **dataset.**
(PDF)
