# Supplementary material for: ObStruct: A Method to Objectively Analyse Factors Driving Population Structure Using Bayesian Ancestry Profiles
Source: PLoS One. 2014 Jan 9;9(1):e85196. doi: 10.1371/journal.pone.0085196 (PMC3887034; doi:10.1371/journal.pone.0085196)
Supplement: Table S1 — Pairwise matrix of values between continents for the human dataset. (PDF) [file pone.0085196.s001.pdf]

Table S1

	Africa	Europe	Oceania	Central South Asia	East Asia	Middle East	America
Africa	0	0.92	0.87	0.82	0.86	0.84	0.54
Europe	0.92	0	0.83	0.29	0.84	0.23	0.52
Oceania	0.87	0.83	0	0.60	0.63	0.74	0.25
Central South Asia	0.82	0.29	0.60	0	0.69	0.30	0.45
East Asia	0.86	0.84	0.63	0.69	0	0.82	0.55
Middle East	0.84	0.23	0.74	0.30	0.82	0	0.52
America	0.54	0.52	0.25	0.45	0.55	0.52	0

*** All values are significant at $p<0.001$

Table S1: Pairwise matrix of R^2 values between continents for the human dataset.
