# Supplementary material for: ObStruct: A Method to Objectively Analyse Factors Driving Population Structure Using Bayesian Ancestry Profiles
Source: PLoS One. 2014 Jan 9;9(1):e85196. doi: 10.1371/journal.pone.0085196 (PMC3887034; doi:10.1371/journal.pone.0085196)
Supplement: Table S2 — Pairwise matrix of values between regions for the Saccharomyces cerevisiae dataset. (PDF) [file pone.0085196.s002.pdf]

Table S2

	West Auckland	Hawkes Bay	Waiheke Island
West Auckland	0	0.20	0.21
Hawkes Bay	0.20	0	0.10
Waiheke Island	0.21	0.10	0

*** All values are significant at p<0.001

Table S2: Pairwise matrix of R^2 values between regions for the *Saccharomyces cerevisiae* dataset.
